# PD-1/PD-L1 binding studies using microscale thermophoresis

**DOI:** 10.1038/s41598-017-17963-1

**Published:** 2017-12-15

**Authors:** Romain Magnez, Bryan Thiroux, Solenne Taront, Zacharie Segaoula, Bruno Quesnel, Xavier Thuru

**Affiliations:** 1 Univ. Lille, UMR-S 1172 - JPArc - Centre de Recherche Jean-Pierre AUBERT Neurosciences et Cancer, F-59000 Lille, France; 2 Inserm, UMR-S 1172, F-59000 Lille, France; 3 CHU Lille, Service des maladies du sang, F-59000 Lille, France

## Abstract

The characterization of protein interactions has become essential in many fields of life science, especially drug discovery. Microscale thermophoresis (MST) is a powerful new method for the quantitative analysis of protein-protein interactions (PPIs) with low sample consumption. In addition, one of the major advantages of this technique is that no tedious purification step is necessary to access the protein of interest. Here, we describe a protocol using MST to determine the binding affinity of the PD-1/PD-L1 couple, which is involved in tumour escape processes, without purification of the target protein from cell lysates. The method requires the overexpression of fluorescent proteins in CHO-K1 cells and describes the optimal conditions for determining the dissociation constant. The protocol has a variety of potential applications in studying the interactions of these proteins with small molecules and demonstrates that MST is a valuable method for studying the PD-1/PD-L1 pathway.

## Introduction

The implications of the immune system for tumour control are well known^1^. While the immune system attempts to neutralize malignant cells, some of the cells persist in a state of equilibrium between their elimination by the immune system and their proliferation. Indeed, tumour cells have developed immuno-evasion mechanisms that allow them to remain in equilibrium with the host by overexpressing immunoregulatory molecules such as PD-L1 and B7-1^2^.

The tumour environment is highly immunosuppressive and locally regulates the anti-tumour response of T lymphocytes infiltrating the tumour. Generally, tumour-infiltrating T lymphocytes express PD-1, and tumour cells express PD-1 ligands (PD-L1 and/or PD-L2)^3^. Blocking the interaction between PD-1 and its ligand using an anti-PD-1 antibody enables restoration of this loss of immune function in cancers of the oropharynx^4^. Thus, some patients may benefit from a more targeted therapeutic approach that blocks the interaction of PD-1 with its ligands^4^. These observations have led to various clinical trials aiming to block the interaction between PD-1 and PD-L1 using the monoclonal antibodies anti-PD-1 or anti-PD-L1 in several types of cancers (melanoma, gastric cancer, multiple myeloma)^5^. Several therapeutic approaches that target the PD-1/PD-L1 blocking pathway are currently being developed.

PD-1 is a type I transmembrane glycoprotein composed of 288 amino acids. It belongs to the immunoglobulin superfamily, which also includes the CD28 and CTLA-4 molecules. PD-1 consists of an extracellular domain, a transmembrane domain and a cytoplasmic domain. The cytoplasmic domain possesses two tyrosines, one in the immunoreceptor tyrosine-based inhibitory motif (ITIM) and the other in the immunoreceptor tyrosine switch motif (ITSM), which are essential for the inhibitory role of the protein. PD-1 is expressed on the surface of CD4+ cells, CD8+ cells, NK cells, B cells, peripheral T cells, dendritic cells and monocytes^6^. A low level of PD-1 is sufficient to ensure the inhibitory role of T-cell activation. In the case of PD-1 deficiency, the administration of PD-L1 does not inhibit the activation of T cells. This lack of inhibition confirms that the inhibitory function of PD-L1 is tightly connected to its interaction with PD-1^7^.

The PD-1 protein interacts with two known ligands: programmed death ligand 1 (PD-L1, B7-H1, CD274) and programmed death ligand 2 (PD-L2, B7-DC, CD273). The PD-L1 molecule is a type I transmembrane protein with 290 amino acids. It is encoded by the CD274 gene, which is found on chromosome 19 in mice and on chromosome 9 in humans. This protein consists of an extracellular domain, a transmembrane domain and an intracellular domain^8^. PD-L1 is expressed on B and T lymphocytes, macrophages, mesenchymal cells and dendritic cells^9^. Unlike other members of this family, PD-L1 is also present on the surface of non-haematopoietic cells such as endothelial, cardiac, pulmonary, pancreatic, muscular and placental cells. The PD-L1 protein was discovered in 1999 as a new member of the B7 molecule family^10^ and was identified as the first known PD-1 ligand the following year^11^. PD-L1 and B7–1 both interact via their IgV-like domain^11^.

Various therapeutic approaches that target the blocking of the PD-1/PD-L1 axis are currently being developed. Blocking receptors on immune effector cells can help eliminate cancer, as described recently with regard to clinical trials evaluating ipilimumab, a blocking antibody of the CTLA-4 receptor, which was accepted as an anti-cancer treatment in 2011^12^. Nivolumab, an antibody targeting the PD-1/PD-L1 pathway, has also recently gained FDA approval based on its significant tumour response in clinical trials for the treatment of melanoma^13^.

Low-molecular-mass inhibitors have also been studied for their ability to modulate the PD-1/PD-L1 signalling pathway. These inhibitors are based on different types of molecules, such as macrocyclic peptides or oxadiazole derivatives^14^. Bristol-Myers Squibb has also recently shown that new PD-L1 inhibitors ((2-methyl-3-biphenylyl) methanol derivatives) under patent can bind to PD-L1 and dissociate the PD-1/PD-L1 complex at stoichiometric concentrations. However, these compounds induce the dimerization of hPD-L1 in solution^15^.

The binding interactions of PD-1 with PD-L1 have been studied by surface plasmon resonance (SPR) and isothermal titration calorimetry (ITC) over the years, but these investigations led to controversy regarding the values of the dissociation constant (K_d_). Equilibrium binding assays were performed to determine the K_d_ between murine PD-1 and PD-L1 and yielded a 4 µM dissociation constant^16^. The cross-species protein interaction of murine PD-L1 and human PD-1 has also been reported in a few studies^17^. In 2007, the equilibrium binding responses between PD-1 and immobilized PD-L1 or PD-L2 were measured by SPR, and a 0.77 µM dissociation constant was obtained for the binding of PD-1/PD-L1^18^. More recently, PD-1 ligands were once again characterized using SPR-based assays, and the results gave a K_d_ of 8 µM for the binding of hPD-1 to hPD-L1 and a K_d_ of 29 µM for the binding of mPD-1 to mPD-L1^19^.

MST is a powerful new method for the quantitative analysis of protein-protein interactions (PPIs) with low sample consumption^20^. The technique is based on the motion of molecules along microscopic temperature gradients, and it detects changes in their hydration shell, charge or size^21^. One binding partner is fluorescently labelled, while the other binding partner remains label-free. We used a protocol that allows the determination of binding affinity by MST without purification of the target protein from the cell lysate^22^. The application of this MST method to PD-1-eGFP and PD-L1-eGFP expressed in CHO-K1 cells allowed us, for the first time, to determine the affinity of the complex formed between PD-1 and its ligand PD-L1 in tumour escape. The protocol has a variety of potential applications for studying the interactions of proteins with small molecules.

## Results

### Thermophoresis is dominated by buffer dependence

To determine the best formulation for analysis of the interaction between a protein and its ligand, several lysis buffers and several buffers for the analysis of thermographs during thermophoresis were studied. The lysis buffer modulates the fluorescence of the labelled protein. As shown for the PD-1-eGFP protein lysis, the use of a buffer without detergents (Table 1) results in reduced fluorescence compared to what is obtained using a detergent-based buffer (Table 1). A protease inhibitor cocktail is also added to prevent degradation of the protein of interest. A radio immunoprecipitation assay (RIPA) buffer (1800 FI units) produces the highest results after extraction. The ideal buffer must provide uniform fluorescence among the sixteen capillaries with a maximum tolerance of 10% from the average. Thermographs should not present the characteristic bumpy curve of aggregation, which hinders the interpretation of measurements.

**Table 1:** Fluorescence counts of PD-1-eGFP proteins in the presence of different lysis buffers. RIPA buffer provides higher fluorescent counts, allowing further dilution of the protein if necessary. Lysis buffer 2 also provides a sufficient amount of fluorescence for microscale thermophoresis. Lysis buffer 1 provides less than 100 FI units and is thus not appropriate for use in thermophoresis.

Buffers	Composition	Proteins	Fluorescence counts
RIPA buffer	25 mM Tris HCl; 150 mM NaCl; 1% NP-40; 0.1% SDS; 1% Sodium desoxycholate	PD-1-eGFP	1800
Lysis buffer 1	25 mM Tris HCl	PD-1-eGFP	80
Lysis buffer 2	20 mM Tris HCl; 130 mM NaCl; 1% NP-40	PD-1-eGFP	1400

Cells expressing the respective fusion proteins PD-1-eGFP and PD-L1-eGFP were both lysed in RIPA buffer. The lysate after extraction was diluted to obtain an optimum fluorescence level between 400 and 1600 FI units. The concentration of the fluorescent protein was kept constant at 35 nM. CHO-K1 cells were used to evaluate the fluorescent background signal, which is undetectable in undiluted lysates. The background signal could be much more significant than in other expression systems and should be monitored. The first measurements were performed at 22 °C in standard capillaries in HEPES buffer^23^ with 60% MST power and 40% LED power (see Supplementary Fig. S1). The fluorescence varied considerably throughout the range (see Supplementary Fig. S1a). The thermographs presented the characteristic bumpy curve of aggregation (see Supplementary Fig. S1b). The background signal was important, as there was a variation in fluorescence between points for the basal phase, which should not occur with an ideal buffer (see Supplementary Fig. S1c). Therefore, the fitted binding curve was biased by the non-ideal operating conditions, which needed to be optimized. The GFP control protein, on the other hand, produced no binding curve with PD-L1 (see Supplementary Fig. S1c). Thus, it was beneficial to perform new experiments using a different buffer to reduce the background signal and the overall fluorescence variation between the different capillaries. SSB buffer^24^ was also investigated with our two proteins of interest. The initial fluorescence of all sixteen capillaries was above the maximum tolerance of 10% from the average (see Supplementary Fig. S1d) but was nonetheless better than the previous fluorescence observed with HEPES buffer (see Supplementary Fig. S1a). The thermographs in SSB buffer (see Supplementary Fig. S1e) did not exhibit bumpy curves; however, no binding curve could be fitted (see Supplementary Fig. S1f). Indeed, the negative control presented the same results as the curve related to the interaction between mPD-1-eGFP and mPD-L1. Therefore, the SSB buffer seems unsuitable for studying the interaction between PD-1 and PD-L1 via thermophoresis.

**Figure 1:**
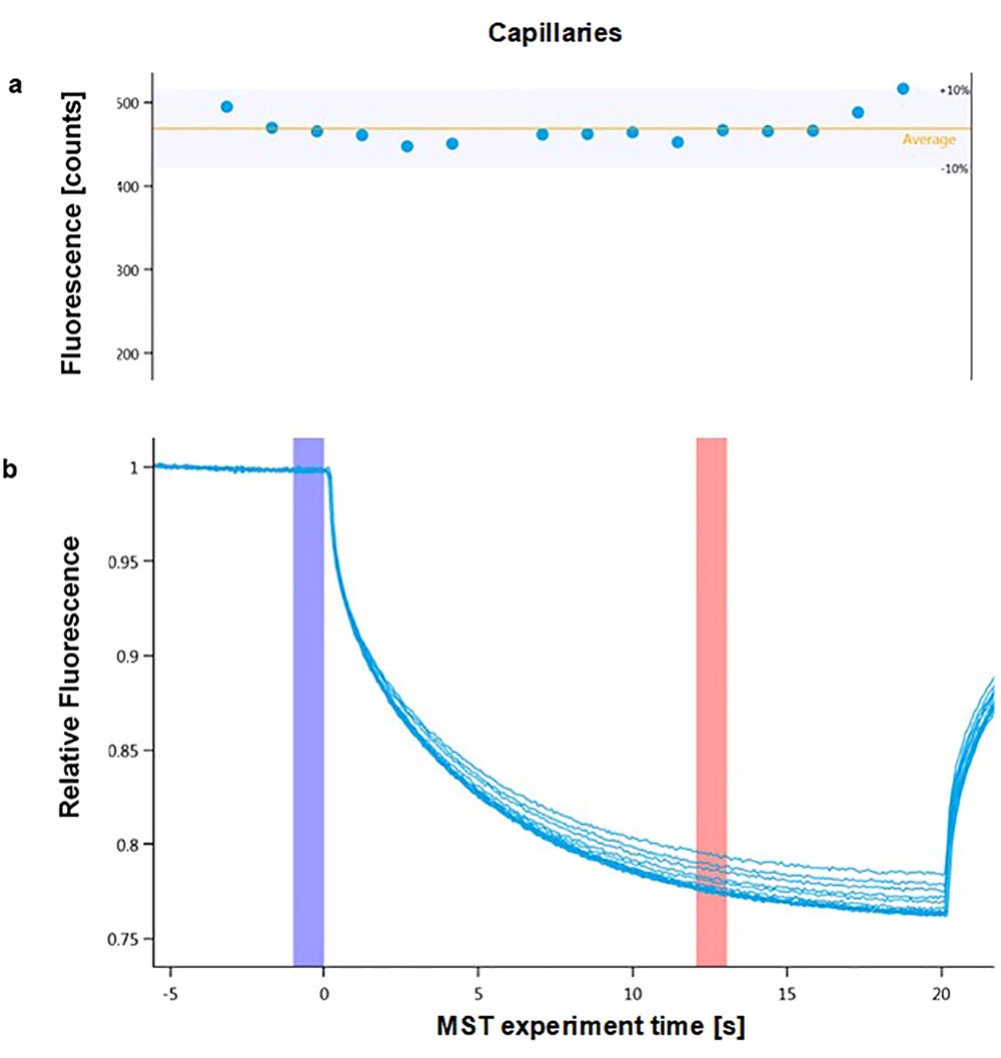
(**a**) Initial fluorescence of PD-1-eGFP in PBS-T buffer at different concentrations of PD-L1. The variation in fluorescence between the different points of the dilution range is within the tolerance range (+/−10%). (**b**) Thermographs of PD-1-eGFP in PBS-T buffer binding to PD-L1 provide well-defined curves. The cold region is set to 0 s (blue) and the hot region to 12 s (red) to determine the K_d_ of the interaction and to avoid potential convection phenomena.

**Figure 2:**
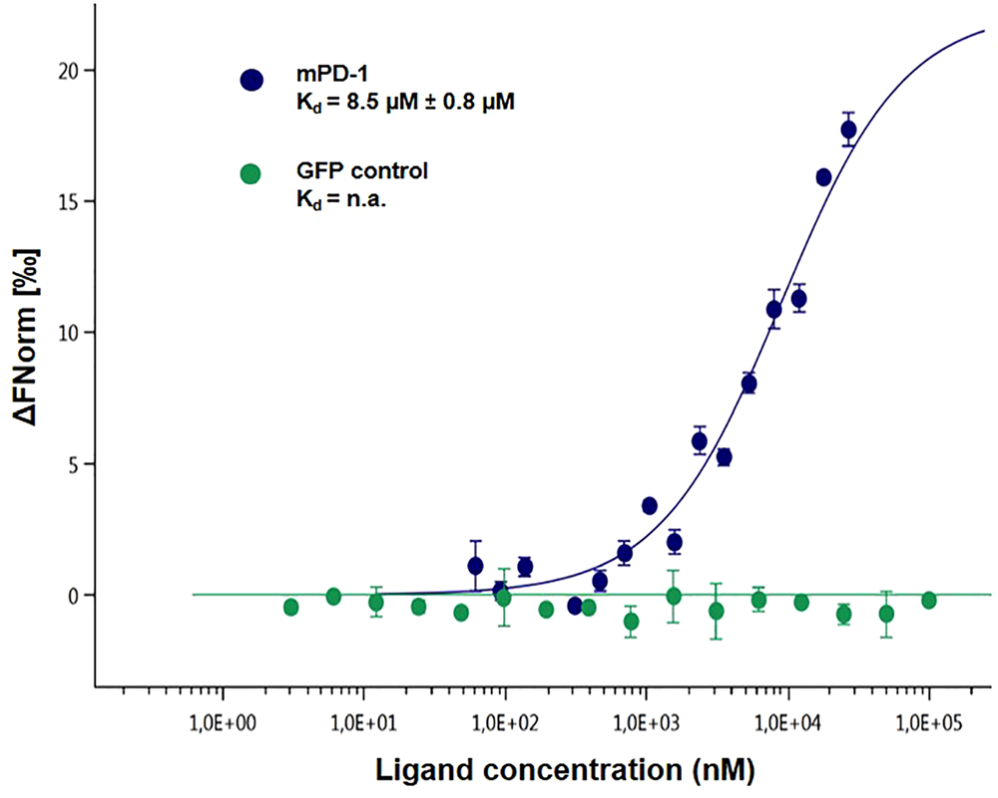
2:1 Dose-response curve for the binding interaction between mPD-L1 and mPD-1-eGFP lysate. The concentration of PD-1-eGFP proteins is kept constant at 35 nM, while the PD-L1 concentration varies from 24 µM to 0.7 nM (dark blue). The binding curve yields a K_d_ of 8.5 µM K_d_. The negative control did not produce a binding curve (green).

PBS-T buffer^25^ was also studied and produced repeatable binding curves that could be fitted. The initial fluorescence of the various capillaries was within the tolerance threshold (Fig. 1a). The thermographs showed no signs of adhesion or aggregation (Fig. 1b). The binding curve relating the ligand concentration to the normalized fluorescence could be fitted (Fig. 2). Indeed, the background signal was reduced compared with those in the previous experiments, and the total amplitude of the signal was higher than the typical requirements, as the amplitude was at least 3-fold greater than the noise level. Thus, the PBS-T buffer seems to be the most suitable buffer to study the interaction of PD-1 with its ligand. The same experiments were conducted with MST 1X buffer and produced similar results to those in PBS-T buffer but with slightly more noise (see Supplementary Fig. S4). The PBS-T buffer was thus retained for further investigations. For every measurement, two negative controls were performed. PD-1-eGFP and PD-L1-eGFP lysates were both tested to verify the absence of binding with a non-relevant protein, and PD-1 and PD-L1 recombinant proteins were tested to verify the absence of binding with a non-relevant eGFP-fused protein. Neither control produced a binding curve.

### Measurement of ligand binding constants

**Figure 3:**
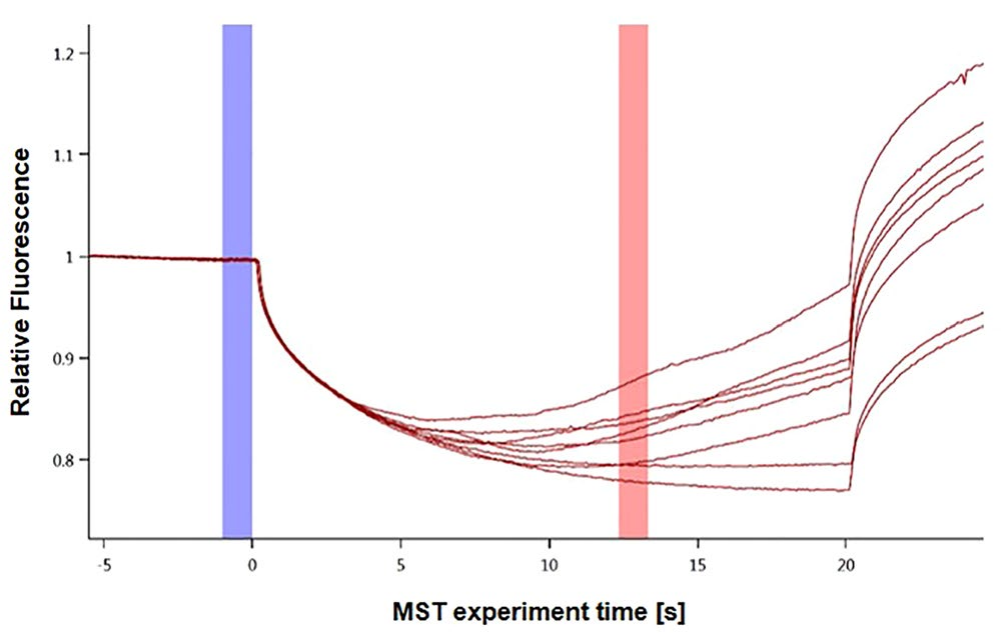
Thermographs of PD-1-eGFP in PBS-T buffer binding to PD-L1 at 37 °C. After 10 s of IR laser heating (MST power: 20%), a very large convection phenomenon was observed. The cold region is set to 0 s (blue) and the hot region to 12 s (red). The disturbance due to this event prevents the proper determination of a dissociation constant.

Additional experiments were performed to determine the best temperature for studying the interaction, as the majority of the highest K_d_ values established by SPR occur at 37 °C^18,19^. On the other hand, most of the published experiments using thermophoresis for interaction studies were conducted between 22 °C^26^ and 25 °C^27^. The binding curves at 37 °C in PBS-T buffer induce a convection phenomenon within the capillaries that makes it impossible to determine K_d_ (Fig. 3). It is therefore preferable to avoid any phenomenon that might disrupt the affinity measurement, which is why a lower temperature was used to study the interaction and to determine the dissociation constant corresponding to the maximum affinity.

After investigating both the ideal MST power for the PBS-T buffer to study the interaction between PD-1 and the PD-L1 ligand and the appropriate concentration range, the experiment was repeated. Each point represents the mean of three sets of measurements. Two different ranges of concentrations were examined in the development of the study of this protein interaction. The first concentration range, which allowed us to define the ideal range, was performed using 1:1 dilutions. To obtain a more defined curve, we opted for 2:1 dilutions, which showed the shift more clearly. Although the curve does not have a perfectly defined saturation phase due to the limited solubility of the ligand described by the supplier, it is possible to determine the K_d_ between our two proteins. The binding of mPD-1-eGFP with mPD-L1 in PBS-T buffer had an affinity of 8.5 μM ± 0.8 μM (Fig. 2). In addition, the GFP control protein exhibited no significant binding with mPD-L1 (Fig. 2).

**Figure 4:**
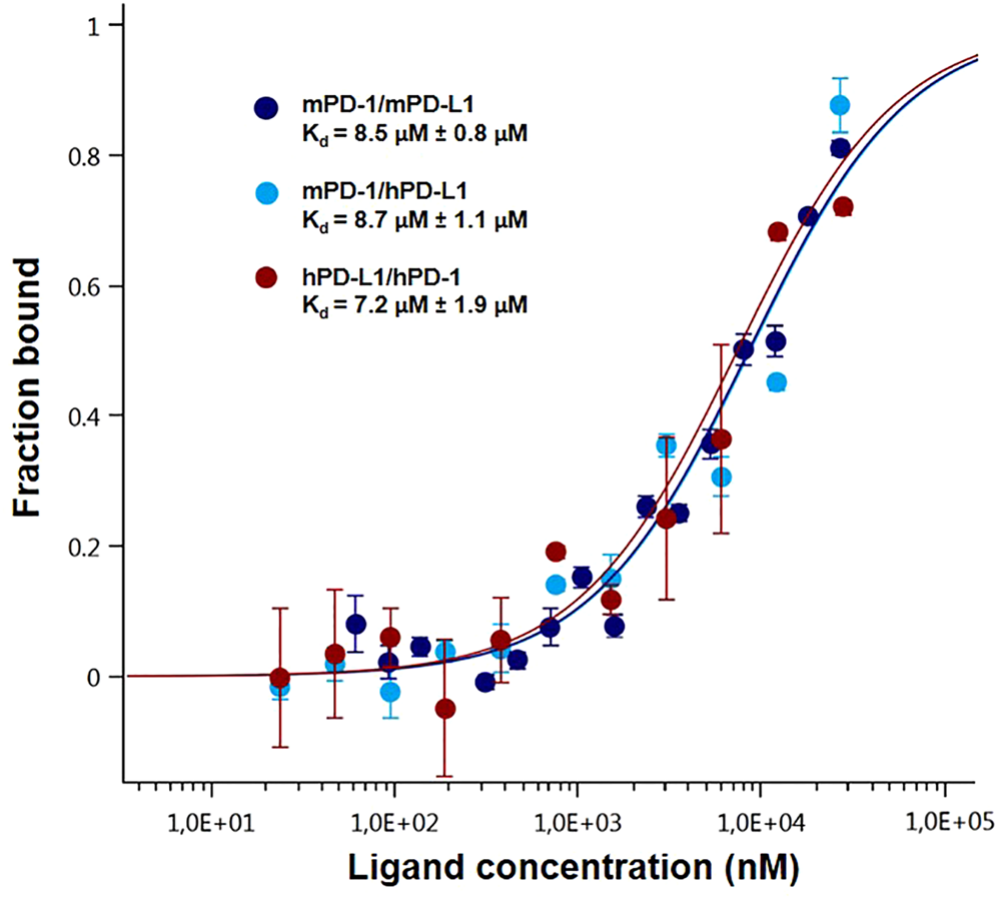
Dose-response curves for the binding interaction between PD-1 and PD-L1. The concentration of PD-1-eGFP or PD-L1-eGFP proteins is kept constant at 35 nM, while the ligand concentration varies from 24 µM to 0.7 nM. All three curves are shown as the fraction bound against ligand concentration to show their similarity, regardless of the variation of the protein under study. The highest affinity is observed for the human PD-1/PD-L1 interaction (brown), which has a 7.2 µM K_d_, whereas the murine variation (dark blue) has an 8.5 µM fitted K_d_. The lowest affinity is observed for the cross-species interaction, which has an 8.7 µM dissociation constant (light blue).

**Figure 5:**
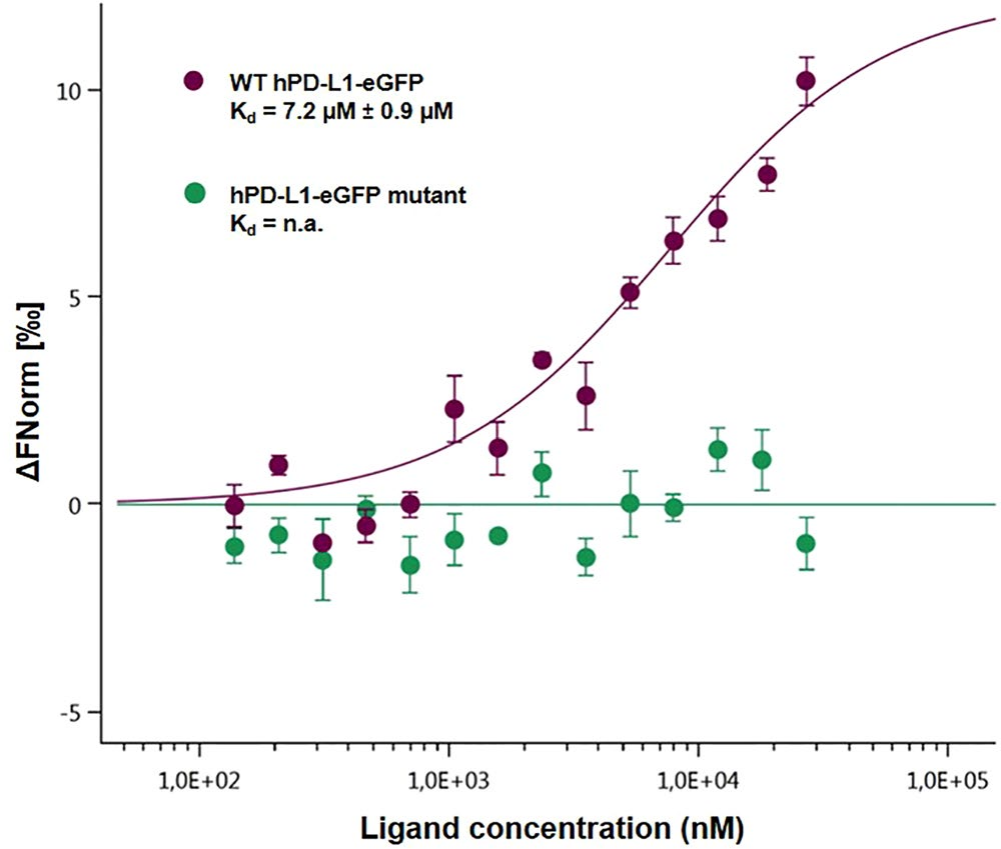
Dose-response curves for the binding interaction between PD-1/hPD-L1-eGFP and its mutant. The concentration of hPD-L1-eGFP and hPD-L1-eGFP mutant proteins is kept constant at 35 nM, while the ligand concentration varies from 24 µM to 0.7 nM. The binding is lost for the mutant protein (green), leading to a flat curve while the binding is maintained, as described previously (7.2 µM) with the wild-type protein (purple).

The overall protocol was also used to monitor the opposite interaction: the interaction of hPD-L1-eGFP with mPD-1 resulted in a calculated K_d_ of 8.7 μM ± 1.1 μM (Fig. 4). Furthermore, hPD-1 binding to hPD-L1-eGFP resulted in an affinity of 7.2 μM ± 1.9 μM (Fig. 4). The final control was performed using PBS-T buffer to monitor the binding between hPD-1 and the wild-type hPD-L1-eGFP protein in one hand, and an hPD-L1-eGFP deletion mutant in the other hand (Supplementary Information 1). The binding is completely lost with the hPD-L1-eGFP deletion mutant protein (Fig. 5). The MST results are in good agreement with previous SPR data revealing a dissociation constant of 8 µM for the binding of PD-1 with PD-L1^19^. These results underline the ability to study biomolecular interactions in highly crowded environments such as cell lysates.

## Discussion

The identification of compounds that might be able to modulate the affinity of the complex formed between PD-1 and PD-L1 represents a major strategy in the development of new treatments targeting immune escape in oncology. Indeed, the identification of small inhibitors that can block the interaction could lead to a more effective immune response. For this reason, a microscale thermophoresis protocol was developed. This protocol involves the overexpression of eGFP fusion protein, which is then extracted from the cell lysate, enabling the determination of the affinity constant between PD-1 and PD-L1 without any purification steps. Thus, the development of this protocol required the production of eGFP fusion proteins. This protocol was designed to quantitatively accelerate the characterization of interactions by avoiding tedious purification steps.

The different observations linked to the thermophoresis curves emphasize the importance of examining various thermophoresis buffers to obtain the optimal conditions, including the best parameters or formulation, to study the affinity of a protein for its ligand. The results suggest that it is essential to minimize the background signal at the different points to achieve precise characterization of the interaction under study and to ensure that the binding observed is not an artificial binding interaction due to an inadequate buffer. Since affinities are highly dependent on the composition of the surrounding matrix, performing experiments in sterically hindered solutions imposes different technical difficulties, mainly due to the potential viscosity and increased background signal. The work reported herein, as well as recent data from the literature, confirmed that the interaction between PD-1 and PD-L1 occurs at a much higher K_d_ than some of the previously reported values^16–18^.

The determination of K_d_ by microscale thermophoresis using eGFP-fused proteins revealed an 8 µM dissociation constant, regardless of the species of protein studied and regardless of which partner is labelled with a fluorescent tag. The use of unpurified fusion proteins from cells overexpressing eGFP proved to be convenient for the quantification of binding affinities for many proteins and types of ligands. This protocol could also be used to perform high-throughput screening with PD-1/PD-L1 inhibitors to evaluate the ability of these recently developed molecules to modulate the affinity of the protein for its ligand, as the small-molecule “blocking pathway” represents a major challenge in the development of new strategies targeting immune escape in oncology.

## Methods

### Cell culture

CHO-K1 cells (ATCC® CCL-61) were cultured in Ham’s F-12 Nutrient Mixture (F12, Life Technologies) with 1% PS (Penicillin-Streptomycin, Life Technologies) and 10% FBS (Foetal Bovine Serum, Life Technologies). Cell lines were cultured at 37 °C in a 5% CO_2_ atmosphere. Cells were passaged every 3 days upon reaching confluence, and the absence of mycoplasma contamination was verified (MycoAlert^TM^ Detection Kit, Lonza).

### Lysis buffer

Several lysis buffers, including some containing detergents, were tested for their extraction efficiency (Supplementary Table S1). A sonication step can be added to optimize protein extraction.

### MST Buffer

Various buffer solutions were used to determine which ones led to the best stability and homogeneity within capillaries when processing microscale thermophoresis experiments: MST buffer (50 mM Tris HCl; 150 mM NaCl; 10 mM MgCl_2_; 0.05% Tween-20), HEPES buffer (50 mM HEPES; 25 mM MgCl_2_; 50 mM NaCl; 0.25% NP-40), SSB buffer (20 mM HEPES; 300 mM NaCl; 0.05% Tween-20), and PBS-T buffer (PBS 1X; 0.05% Tween 20).

### Microscale thermophoresis requirements

The quantity of cells for this protocol can vary from 10 to 40 million. The optimal fluorescence of lysate is situated between 200 and 1600 units of fluorescence (FI units). Different types of additives such as Tween 20, various MgCl_2_ concentrations, and BSA have been tested to prevent sample aggregation, and different types of capillaries have been investigated to reduce the adsorption within capillaries. Various buffers have also been tested and are listed in the relevant section. The fluorescence is measured using a small quantity of cell lysate within a capillary chosen from different types of available capillaries: standard, premium, hydrophilic or hydrophilic. The highest concentration of ligand must be at least 20 times higher than the expected dissociation constant of the interaction under study. However, the lowest concentration of ligand must be lower than the concentration of the fluorescent protein. An online tool created by NanoTemper® can be used to determine the ideal concentration range. The concentration of the fluorescent protein can be determined using a GFP dosage kit (Abnova KA0911) or a calibration curve (Supplementary Information 2).

### MST assay optimization

MST experiments were performed on a NanoTemper ® Monolith NT.115 with blue/red filters (NanoTemper Technologies GmbH, Munich, Germany). Samples were prepared in the different buffers listed in the MST buffer section and loaded into standard/premium treated capillaries. Measurements were performed at 22 °C using 20% MST power with laser off/on times of 5 s and 30 s, respectively, and 60% MST power with laser off/on times of 5 s and 20 s. All experiments were repeated three times for each measurement. Data analyses were performed using the NanoTemper® analysis software. The K_d_ constants between a protein and its ligand can be calculated using the saturation binding curve at equilibrium^27^. The fitting function is derived from the law of mass action:

1 $$\frac{[LP]}{[{P}_{{\rm{t}}{\rm{o}}{\rm{t}}}]}=\frac{[{L}_{tot}]+[{P}_{tot}]+{K}_{d}-\sqrt{{([{L}_{{\rm{t}}{\rm{o}}{\rm{t}}}]+[{P}_{{\rm{t}}{\rm{o}}{\rm{t}}}]+[{K}_{d}])}^{2}-4[{L}_{{\rm{t}}{\rm{o}}{\rm{t}}}].[{P}_{{\rm{t}}{\rm{o}}{\rm{t}}}]}}{2[{P}_{{\rm{t}}{\rm{o}}{\rm{t}}}]}$$

*[LP]:* Bound complex concentration

*[P*_*tot*_ *]:* Total protein concentration

*[L*_*tot*_ *]:* Total ligand concentration

*K*_*d*_: Dissociation constant

### Plasmids

A pcDNA3.1 plasmid containing an mPD-1-YFP fused protein was purchased from Life Technologies (PD-1: 870 bp, Glycine-alanine-linker: 42 bp, YFP: 732 bp). The pcDNA3.1 Hygro plasmid was also purchased from Life Technologies. The plasmid was digested by two restriction enzymes, HindIII and EcoRI (New-England-Biolabs 2000), to allow the insertion of our mPD-1-YFP construct. The pVITRO eGFP plasmid was purchased from InvivoGen and was used for PCR amplification to obtain the eGFP amplicon. The plasmid containing hPD-L1-eGFP was purchased from GeneCopoeia (CS-GS402L-M10/pReceiver-M10). The plasmid containing the mutant variation of hPD-L1-eGFP was also purchased from GeneCopoeia (CS-GS406L-M10/pReceiver-M10). Both sequences are based on the crystal structure of the PD-1/PD-L1 complex (PDB code: 3BIK) and are described in Supplementary Information 1.

### Primer design

Thermo Scientific webtools were used to design two set of primers, which were purchased from Eurogentec for use in pcDNA 3.1 Hygro constructs. The first set of primers, which flanked the PD-1-linker-YFP gene sequence for insertion into the pcDNA 3.1 Hygro plasmid, was as follows: F: 5′-ATGTGGGTCCGGCAGGTA-3′ and R: 5′-TTATCTGAGTCCGGAGCGGTAC-3′. The second set of primers, which was used to switch the YFP with eGFP, was as follows: F: 5′-GTGAGCAAGGGCGAGGAG-3′ and R: TTACTTGTACAGCTCGTCCATGC (Supplementary Information: Table S2).

### Plasmid digestion

The digestion was performed with 4 units of enzyme per μg of plasmid. The volume of enzymes should not exceed one tenth of the volume of the mixture and must adhere to the proportions given for a digestion mixture (Supplementary Table S1). The digestion was performed for 1 h at the appropriate temperature (37 °C) in a heating block. The digestion products could be analysed on agarose gel. The enzymes and their buffers were purchased from Invitrogen.

### PCR amplification and purification

The primers were diluted to obtain a final concentration of 10 μM (Supplementary Table S3). The DNA polymerase used (Kapa HiFi HotStart Readymix) was provided by CliniSciences. The thermal profile was as follows: initial denaturing step at 95 °C for 3 min, followed by 25 cycles, each consisting of 20 s denaturing at 98 °C, 15 s annealing at 57 °C and 1 min extension at 72 °C, followed by a final extension step at 72 °C for 3 min. The PCR product was then purified using the PCR clean-up gel extraction provided by Machery-Nagel, following the manufacturer’s instructions. The amplicon was sequenced and analysed by GATC Biotech to verify the absence of mutations and the conservation of the reading frame. A schematic of the overall biomolecular process is described in Supplementary Fig. S3.

### mPD-1-eGFP construct

The purified amplicon was then ligated to the digested pCDNA 3.1 Hygro plasmid using the In-Fusion HD Cloning Kit from Clontech Laboratories, following the manufacturer’s instructions. To perform the ligation between the vector and the insert, it was necessary to use a 100 ng/μL vector solution with 50 ng/μL insert. The solution was then incubated for 15 min at 50 °C.

### Bacterial strain and PD-1-eGFP transformation

Mix & Go competent *E*. *coli* cells (Zymo Research) were used for the cloning of DNA fragments and the preparation of plasmids because they possess recA1 and endA1 gene mutations that increase the insert stability and the extracted DNA quality. The strains were stored at −80 °C. The pcDNA3.1 Hygro/PD-1-eGFP plasmid was transformed into the *E*. *coli* cells and then purified using the Mini Plasmid Prep Kit (Plasmid DNA purification, Nucleospin Plasmid, Machery-Nagel). The plasmid concentration was then quantified using a NanoDrop (ThermoFisher Scientific), and the concentration was adjusted to 2 µg/µL to simplify further cell transfection.

### Cell transfection

CHO-K1 cells were transfected with the Cell Line Nucleofector® Kit T, Program U-023 and the pcDNA 3.1 Hygro plasmid expressing PD-1-eGFP. Two micrograms of plasmid was transfected into one million CHO-K1 cells suspended in 100 µL of Nucleofector solution. Cells were analysed by flow cytometry 48 h after Nucleofection®. Cells were then subjected to an appropriate selective pressure that allowed the growth only of cells containing the DNA insert. After three weeks of selective pressure, the multiparametric analysis of transfected CHO-K1 cells overexpressing mPD-1-eGFP allowed the definition of sub-populations that could be separated from the global population. The population of cells strongly expressing eGFP was analysed by flow cytometry and retained for microscale thermophoresis analysis (Supplementary Fig. S2). The identical protocol from transfection to cytometry analysis was also performed on CHO-K1 cells expressing the hPD-L1-eGFP protein.

### Protein extraction

CHO-K1 cells overexpressing R-eGFP proteins (R = PD-1, PD-L1, non-relevant) were treated when they reached 80% confluency in T75 flasks. The cells were washed with 5 mL of PBS and then trypsinized with 1.5 mL of trypsin to avoid using a cell scraper. After the cells were incubated for 5 min, 3.5 mL of complete culture medium as described in the cell culture section was added to the trypsinized cells. Cells were then centrifuged at 1200 rpm at 4 °C for 5 min. The supernatant was removed, and the cells were washed again with 5 mL of PBS and then centrifuged again at 4 °C for 5 min. Once the PBS supernatant was removed, cells overexpressing mPD-1-eGFP were suspended in 300 µL RIPA buffer. The cells were kept on ice to minimize local overheating. Protease inhibitor cocktail (1%) was also added to the mixture (Halt™ Protease Inhibitor Cocktail, EDTA-Free, Thermo Fisher Scientific). The lysate was collected by centrifugation at 15,000 rpm at 4 °C for 45 min to remove large aggregates and cell debris. Strong centrifugation is recommended to reduce the background that can occur in an MST experiment. The lysate was divided into aliquots, which were stored at appropriate temperatures for further investigation. The overall schematic process of this protocol from cell culture to MST measurements is described in Supplementary Fig. S5.

### PD-1 binding assay

MST was used to establish the binding affinity between PD-1-eGFP and PD-L1 (R&D Systems, Inc., Minneapolis, MN). Cell lysate was diluted in PBS-T buffer (0.05% Tween 20) to a final concentration where the fluorescent signals of the GFP proteins were similar and above the typical detection limit of the Monolith NT.115 instrument (NanoTemper Technologies). The final concentration of cell lysate was kept constant at 35 nM. For the binding between mPD-1-eGFP and h/mPD-L1, 10 µL of the 54 µM PD-L1 solution was diluted 2:1 in 10 µL buffer to make a 16-sample dilution series extending to 61.7 nM. Another concentration range was prepared using a 1:1 dilution from 24 µM to 0.7 nM with the other parameters remaining the same. Ten microliters of cell lysate was added to 10 µL of each ligand solution.

### PD-L1 binding assay

MST was used to establish the binding affinity between PD-L1-eGFP and PD-1 (R&D Systems, Inc., Minneapolis, MN). Cell lysate was diluted in PBS-T buffer (0.05% Tween 20) to a final concentration where the fluorescent signals of the GFP proteins were similar and above the typical detection limit of the Monolith NT.115 instrument (NanoTemper Technologies). The final concentration of cell lysate was kept constant at 35 nM. For the binding between hPD-L1-eGFP with h/mPD-1, 10 µL of the 48 µM h/mPD-1 solution was diluted 1:1 in 10 µL buffer to make a 16-sample dilution series extending to 0.7 nM. Ten microliters of cell lysate was added to 10 µL of each ligand solution.

## Electronic supplementary material

Supplementary Information
