# Supplementary material for: PD-1/PD-L1 binding studies using microscale thermophoresis
Source: Sci Rep. 2017 Dec 15;7:17623. doi: 10.1038/s41598-017-17963-1 (PMC5732298; doi:10.1038/s41598-017-17963-1)
Supplement: Supplementary file 1 — Supplementary Information [file 41598_2017_17963_MOESM1_ESM.pdf]

Supplementary Data

PD-1/PD-L1 binding studies using microscale thermophoresis

Romain Magnez[1,2], Bryan Thiroux[1,2], Solenne Taront[1,2], Zacharie Segaoula[1,2], Bruno Quesnel[1,2,3], Xavier Thuru[1,2*]

[1]Univ. Lille, UMR-S 1172 - JPArc - Centre de Recherche Jean-Pierre AUBERT Neurosciences et Cancer, F-59000 Lille, France

[2]Inserm, UMR-S 1172, F-59000 Lille, France

[3]CHU Lille, Service des maladies du sang, F-59000 Lille, France

***Corresponding author:** xavier.thuru@inserm.fr

Univ. Lille, UMR-S 1172 - JPArc - Centre de Recherche Jean-Pierre AUBERT Neurosciences et Cancer, F-59000 Lille, France

Supplement

Supplementary Tables

Buffers	Composition
RIPA buffer	25 mM Tris HCl; 150 mM NaCl; 1% NP-40; 0.1% SDS; 1% sodium desoxycholate
Lysis buffer 1	25 mM Tris HCl
Lysis buffer 2	20 mM Tris HCl; 130 mM NaCl; 1% NP-40

Table S1 | Buffer compositions used for the cell lysis of the CHO-K1 cell line.

Reagent	Volume (µL)
10X Buffer	6 µL
Enzyme 1	3 µL
Enzyme 2	3 µL
Vector	7.5 µL
Water	To 60 µL

Table S2 | Composition of the digestion mix.

Reagent	Volume (µL)
Kapa HiFi	12.5 µL
Primer Mix	1.5 µL
Water	To 25 µL

Table S3 | Composition of the PCR mix.

Supplementary Information 1: Schematic presentation of hPD-L1-eGFP and its deletion mutant.

PD-L1 sequences of plasmids purchased from GeneCopoeia (CS-GS402L-M10/pReceiver-M10 and CS-GS406L-M10/pReceiver-M10) are described below. Restriction enzymes sites are highlighted in red.

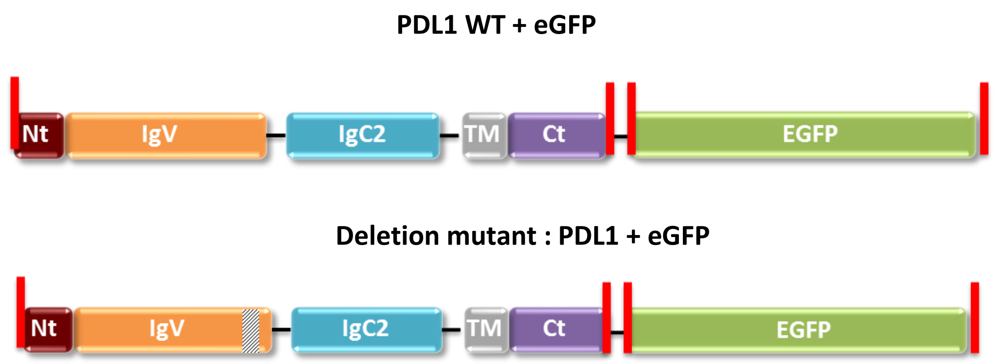


```
PDL1 WT                 51  HGEEDLKVQHSSYRQRARLLKDQLSLGNAALQITDVKLQDAGVYRCMISY   100
                            ||||||||||||||||||||||||||||||||||||||||||
PDL1 Deletion mutant    51  HGEEDLKVQHSSYRQRARLLKDQLSLGNAALQITDVKLQDAG--------    92

PDL1 WT                101  GGADYKRITVKVNAPYNKINQRILVVDPVTSEHELT   136
                                   |||||||||||||||||||||||||||||
PDL1 Deletion mutant    93  -------ITVKVNAPYNKINQRILVVDPVTSEHELT   121
```

Supplementary Information 2: Concentration of the eGFP fusion protein

Calibration curve

The fluorescein calibration curve was obtained on the thermophoresis instrument by establishing a range with different concentration points (from 0 to 100 nM). Each concentration was linked to an amount of fluorescein fluorescence (FI units). The concentration of the labelled protein eGFP was determined using the following relationship, which connects the fluorescein units to the eGFP, their quantum yields, and the respective molar extinction coefficients at a given excitation length:

$$\frac{\text{FI units (fluorescein)}}{\text{FI units (GFP)}} = \frac{\varphi\text{f (fluorescein)} \times \varepsilon(\lambda)\text{fluorescein}}{\varphi\text{f (GFP)} \times \varepsilon(\lambda)\text{fluorescein (GFP)}} \quad (1)$$

Since the FI units (GFP) were provided by cap-scan, it was possible to determine the corresponding FI units (fluorescein) on the calibration curve to determine the concentration of the labelled protein eGFP.

GFP Quantitation Kit

The GFP concentration was quantified using the GFP Quantitation Kit provided by Abnova ®. A GFP standard at 1 µg/µL was diluted to obtain a 10 ng/µL solution. A range was constructed on a 96-well plate using the GFP buffer provided. The concentrations in the wells varied from 0 ng/well to 400 ng/well of standard GFP: 0, 20, 40, 60, 80, 100, 200, 300, and 400 ng/well. A small quantity of the lysate to be measured (1-100 µL) was poured into a well and brought to a volume of 100 µL using GFP buffer. The plate was then read by means of a plate reader and to establish a calibration curve. The concentration of the labelled molecule was determined using the calibration curve of the GFP standard.

Supplementary Fig. S1

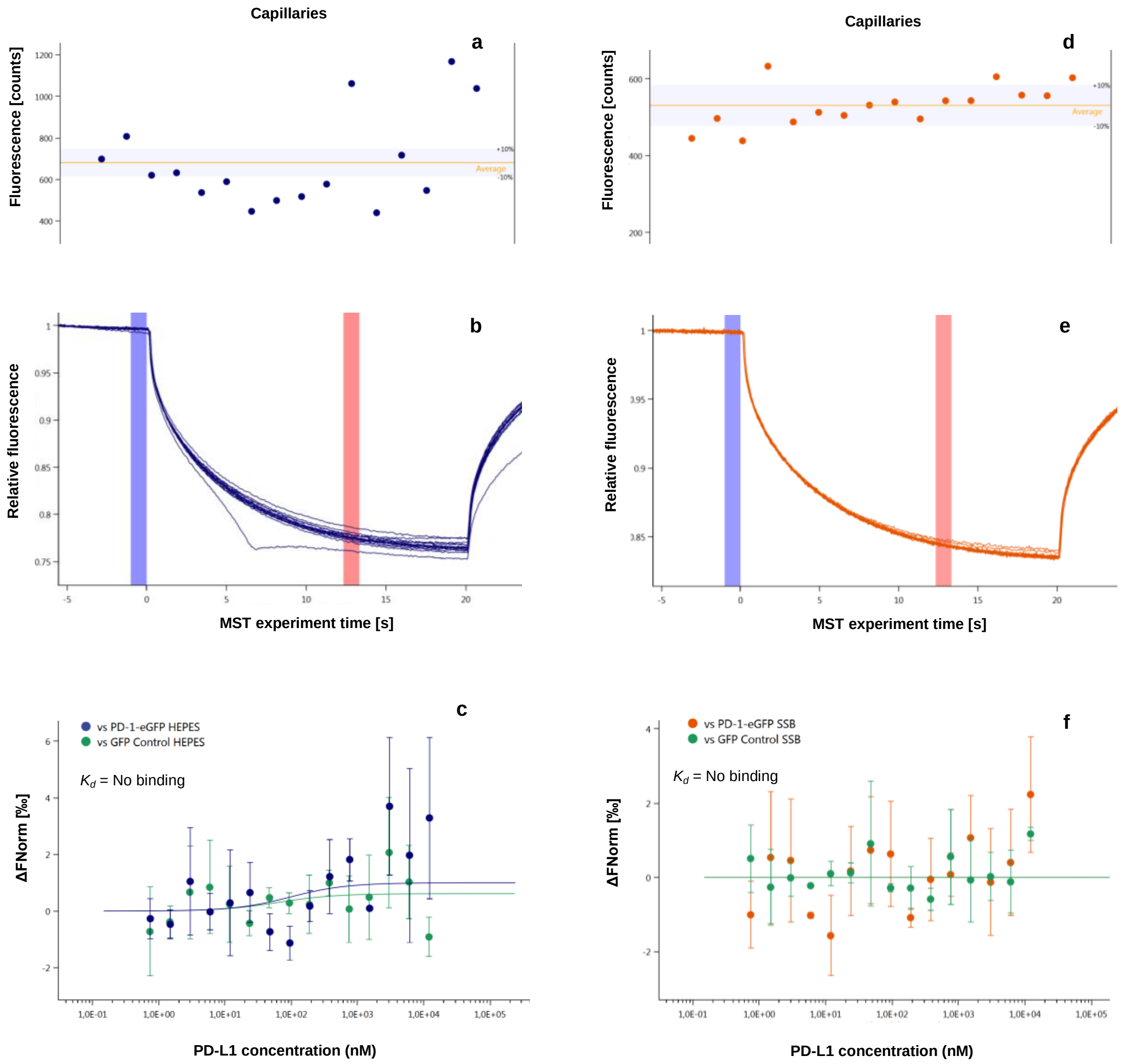

Buffer impact on PD-1 / PD-L1 interaction

(**a**) Initial fluorescence of PD-1-eGFP in HEPES buffer at different concentrations of PD-L1. The fluorescence variation is very high at the different points in the range, which indicates that the curve will not be interpretable. (**b**) Thermographs of PD-1-eGFP binding to PD-L1 in HEPES buffer, which results in large aggregates. Better sample preparation is needed. The cold region is set to 0 s (blue) and the hot region to 12 s (red) to establish the approximate K_d of the interaction and to avoid potential convection phenomena. (**c**) Measurement of PD-1-eGFP binding to PD-L1 in HEPES buffer (blue) at 22 °C. No binding is observed. The negative control (green) did not yield a binding curve. (**d**) Initial fluorescence of PD-1-eGFP in SSB buffer at different concentrations of PD-L1. The fluorescence variation is quite high for some points (>20%), exceeding the fluorescence variation tolerance. The average fluorescence is less noisy than in the HEPES buffer shown above but is still important for a few

points in the dilution range. (**e**) Thermographs of PD-1-eGFP binding to PD-L1 in SSB buffer, resulting in repeatable thermographs. The cold region is set to 0 s (blue) and the hot region to 12 s (red) to establish the approximate K_d of the interaction and to avoid potential convection phenomena. (**f**) the dose-response curve for the binding interaction of our two proteins in SSB buffer (orange) at 22 °C cannot provide a fitted K_d. The negative control (green) did not yield a binding curve.

Supplementary Fig. S2

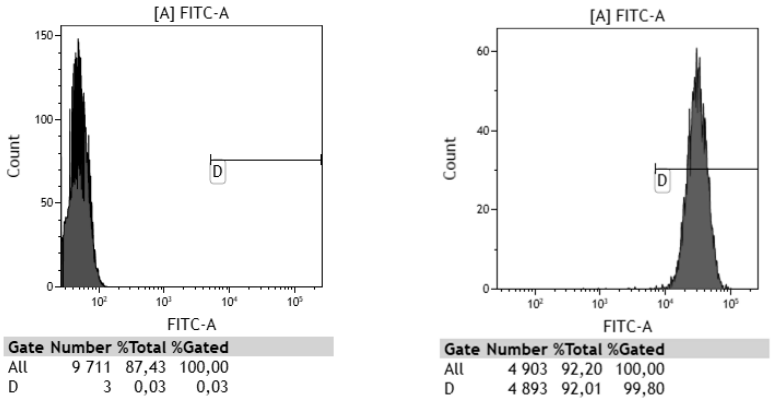

Measurements of eGFP cell fluorescence by flow cytometry.

(a) Fluorescence control of CHO-K1 cells transfected with pcDNA3.1 Hygro. The whole population of cells clearly shows no significant GFP fluorescence. (b) Fluorescence of CHO-K1 cells transfected with pcDNA3.1 Hygro/PD1-eGFP after three weeks of selective pressure. The cell population is clearly expressing our GFP protein (>10^4) with very high yield and purity.

Supplementary Fig. S3

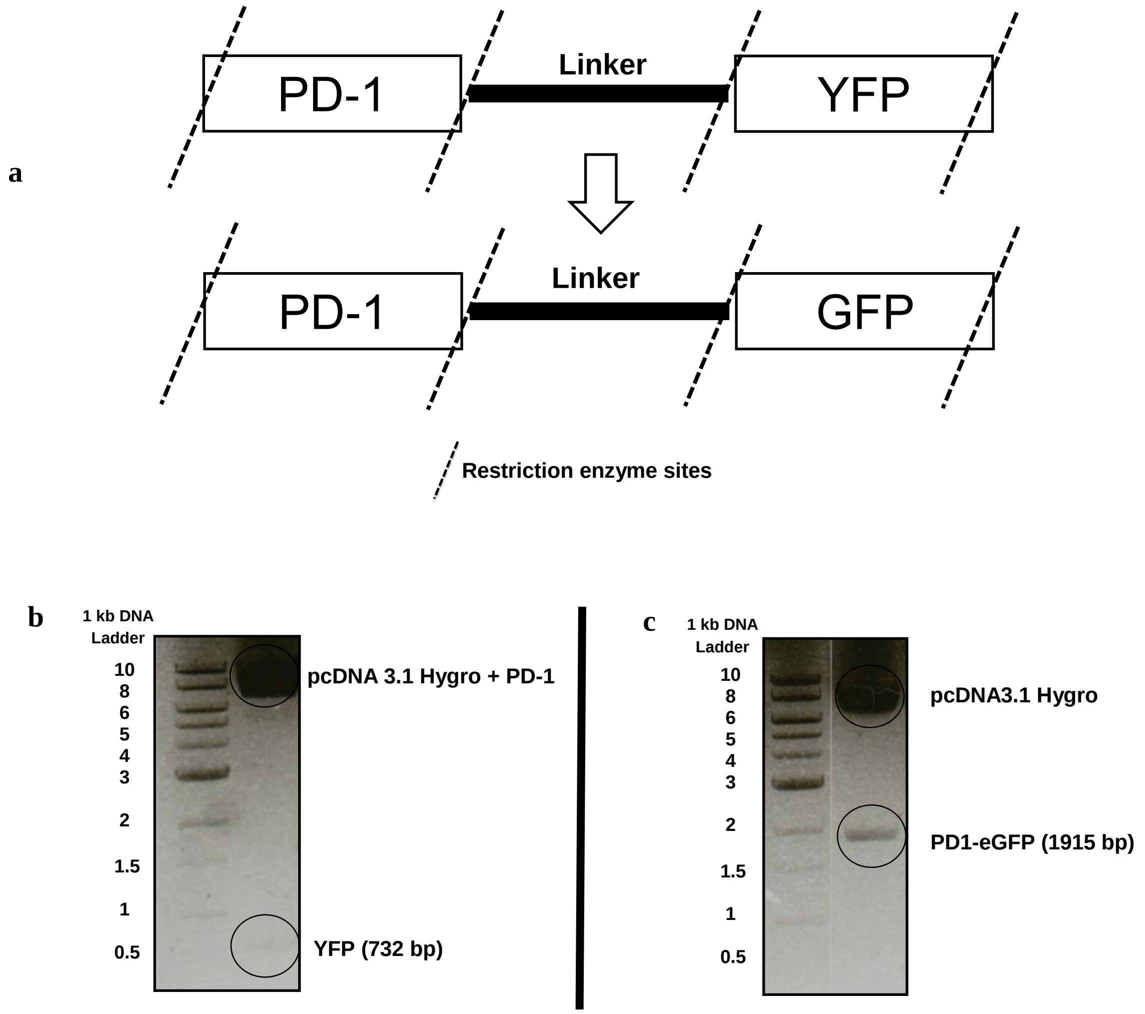

PD-1-eGFP construction strategy.

(a) Summarized biomolecular protocol combining digestion of our pcDNA.3.1/PD-1-YFP plasmid with the appropriate enzymes, PCR amplification, ligation and bacterial amplification to obtain the PD-1-eGFP construct used for the microscale thermophoresis protocol. (b) 1% TAE (40 mM Tris acetate; 2 mM EDTA) agarose gel of the digested pcDNA3.1/PD-1-YFP plasmid. Excision of the YFP produces a band at ~732 bp. (c) 1% TAE (40 mM Tris acetate; 2 mM EDTA) agarose gel of the digested PD-1-eGFP construction leads to a band at ~1915 bp, which was sent for sequencing analysis, allowing us to validate our final construction. A 1 kb DNA Ladder (BioLabs) is used in the gels in both (b) and (c).

Supplementary Fig. S4

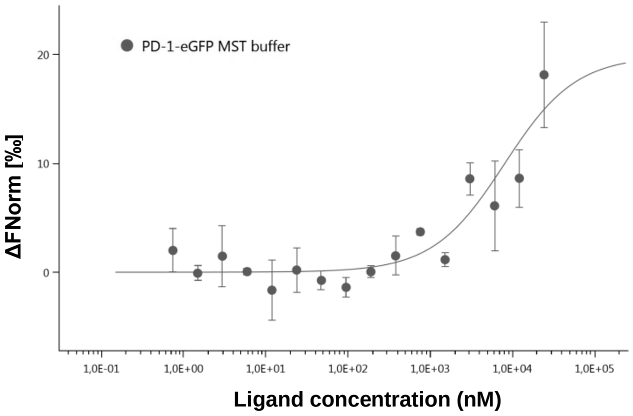

Dose-response curve in MST buffer between PD-1-eGFP and different concentrations of PD-L1, allowing the measurement of K_d at ~ 8 µM.

MST experiments on the PD-1/PD-L1 interaction conducted in MST buffer can produce binding curves that range from satisfactory to very random binding. However, as shown by the curve in this supplementary data, the unbound state phase can be somewhat noisy. In addition, the bound state seems less defined than in PBS-T buffer, and obtaining a precise K_d fit using the software might be difficult, probably due to the ionic strength of this buffer, which might not be appropriate for the protein under study.

Supplementary Fig. S5

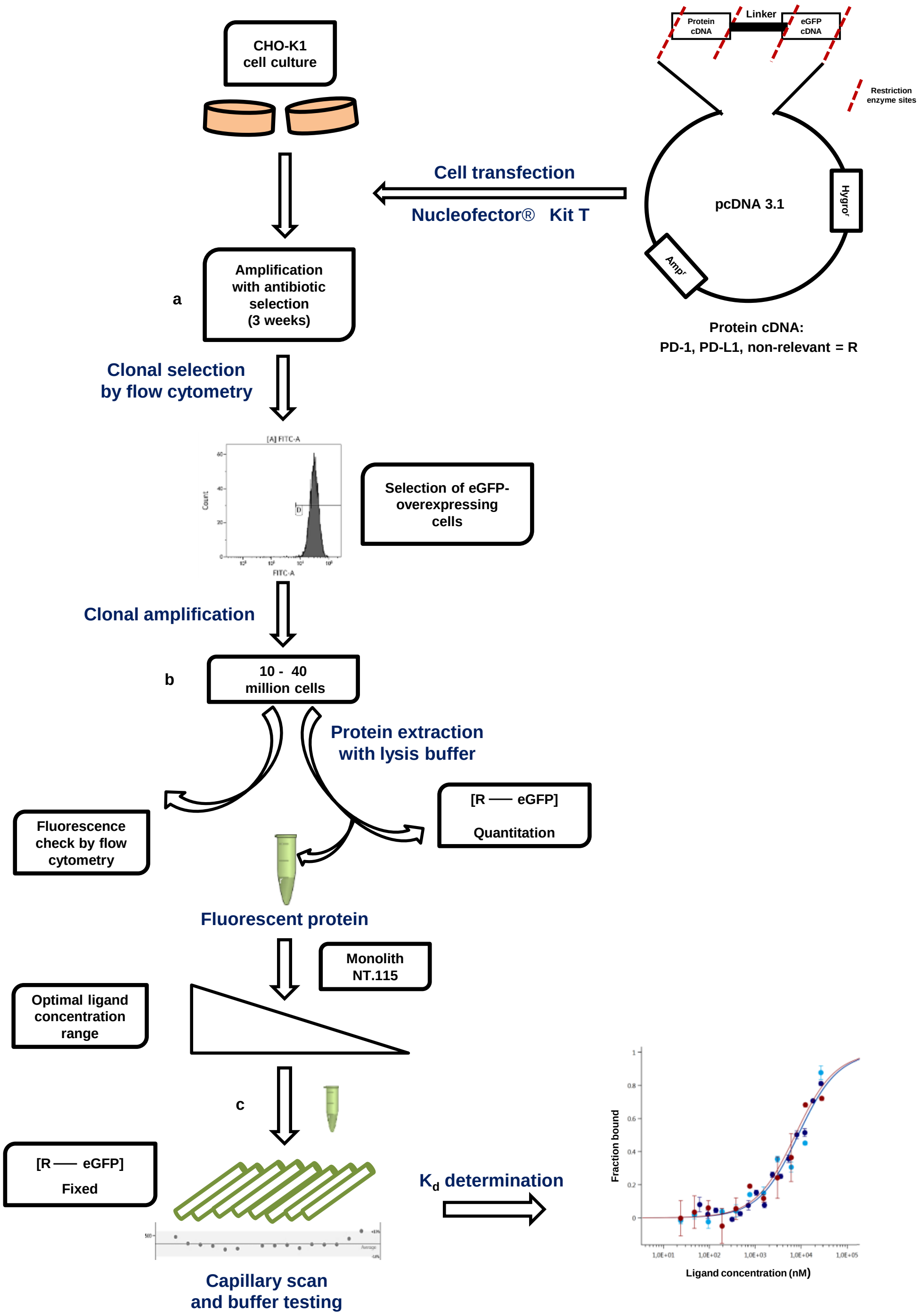

Schematic protocol of the microscale thermophoresis process.

(a) Cell culture followed by transfection with a pcDNA 3.1 plasmid expressing eGFP fusion proteins. (b) GFP-overexpressing cells analysed by flow cytometry are retained for amplification and further protein extraction. (c) MST measurements using optimal ligand concentration range.
